# Annotations

**Published:** 1902-08-16

**Authors:** 


### Annotations.

### The King's Thank-offering.

The minds of kings are not revealed to ordinary man, nor do we presume to suggest what thoughts may have occurred to a royal convalescent while lying on deck on the waters of the Solent; but no one can doubt that the King's offer to the nation of his Osborne estate for the purpose of a Convalescent Home will always be regarded by the people as an expression of gratitude—the King's thank-offering for his happy recovery from a most serious illness. Perhaps in the midst of gorgeous pageants and the accounts thereof we are a little apt to forget how grave was the malady which only a few short weeks ago threatened not only the Coronation but the very life of the Sovereign. May be in all the turmoil in which London has been involved, the hoardings, the "stands," the dislocation of business, and the ignoble quarrelling about "seat money," some may now be glad to settle down to ordinary life once more, and in their gladness may overlook the danger which is past. But the King does not forget; and in offering his palace on the Solent in order that officers of the Navy and Army whose health has been impaired in rendering service to their country may partake of those advantages which have proved so beneficial in restoring his own health and strength, he has signalised his recovery in a manner which is most graceful and acceptable. The ties between the King and his people are many; but no one can doubt that they have been added to by the knowledge that at the beginning of the serious illness from which he has so happily recovered he risked much in order that his people might not be disappointed, and that when the time came he faced danger without flinching. Still more are they added to by the knowledge that now, on his recovery, his first thought has been for others, that they also may be helped back to health.

### "An Infected Area."

Some months ago a letter which attracted considerable attention appeared in the columns of the *Times* advocating as a precautionary measure the closing of the Rowton Houses until the small-pox epidemic had run its course. That the proposal was too sweeping was evident at once. Nevertheless it has to be admitted that there are dangers connected with the aggregation in such close compass night after night of so many people whose occupations are so various and who work in so many quarters. The risk of the dissemination of infection is a very real one, and is obvious at once; the risk of the buildings themselves, or their fittings, or maybe their dust, becoming so infected as to be a danger to their lodgers is not so apparent; but a curious series of cases of small-pox which seems to have occurred on one floor of one of these establishments, suggests the possibility that unless great care be taken caravanserais of this kind might become truly "infected areas." If reports are correct it would appear that a considerable number of cases of small-pox have been removed from the Rowton House, King's Cross, by far the greater number of these cases having occurred on the third floor of the building, this floor, like the other five, being a long corridor having the cubicles on either side. On this third floor we are told there have been altogether since the beginning of the year nearly twenty cases, and the officials have at last adopted the plan of giving every lodger on this particular floor the choice of submitting to vaccination or leaving the place. That the disease should have lurked for month after month in this corridor is a matter of much interest. It has been decided that establishments like the Rowton Houses are not "common lodging houses," and are thus independent of that strict sanitary control which the authorities are able to exercise over such houses, and it becomes very evident that, in view of the large numbers of people accommodated, such sixpenny hotels might become perfect hotbeds of disease, unless they were very carefully managed. We have every confidence that in all buildings owned by "Rowton Houses Limited" everything is done that can be done to lessen risks, but there is nothing to prevent anyone else from entering upon the same business, and it is impossible not to see that, if such an occurrence as that to which we have alluded can take place in a Rowton House with all its guarantees, houses of similar size, but erected without any guarantees at all, might become "infected areas," dangerous to all around, and yet as completely outside the control of the sanitary authorities as is any ordinary "Temperance Hotel."

### The Late Effects of the Roentgen Rays.

It is evident that we are as yet far from having attained to completeness of knowledge in regard to the effects which may be produced by the Roentgen rays. That they may cause very unpleasant and slowly healing "burns" is now well known, as also that these effects may not show themselves for some time after the application. Again, it has been shown that in some cases, even when no ulceration is produced, considerable pigmentation may be set up, so that it may occasionally happen that in the attempt to get rid of a degree of hirsuteness which may be, comparatively speaking, not a matter of great consequence, an amount of pigmentation may be produced which may be most annoying and may last for a long time. Lately, however, another unfortunate result of the rays has come to light, namely, the development of disfiguring telangiectases on the area dealt with.. These starlike dilatations of the blood-vessels when they occur in any number may be very disfiguring, and there is this that is curious about them that in some cases a considerable time—several months—seems to have elapsed before they showed themselves. There can be no doubt that the rays in passing through the tissues exercise a selective power, affecting certain tissues destructively (as is well shown in their action upon certain forms of cancer), and it may be presumed that in the cases mentioned the dilatation of the cutaneous blood-vessels has either been due to interference with the circulation by a form of scar contraction following the partial destruction of certain tissues around the vessels, or may even have been due to the destructive effects of the rays upon the walls of the vessels themselves, leading to their gradual dilatation under the pressure of the blood. But, however they are caused, they serve well to show how careful one must be in the use of an agent the nature of which we do not as yet fully understand.

